# The FUSE binding proteins FBP1 and FBP3 are potential *c-myc *regulators in renal, but not in prostate and bladder cancer

**DOI:** 10.1186/1471-2407-8-369

**Published:** 2008-12-16

**Authors:** Achim Weber, Ilka Kristiansen, Manfred Johannsen, Beibei Oelrich, Katharina Scholmann, Sven Gunia, Matthias May, Hellmuth-Alexander Meyer, Silvia Behnke, Holger Moch, Glen Kristiansen

**Affiliations:** 1 Department of Pathology, Institute of Surgical Pathology, University Hospital Zurich, Zurich, Switzerland; 2 Department of Urology, Charité Campus Mitte, Berlin, Germany; 3 Institute of Pathology, Charité Campus Mitte, Berlin, Germany; 4 Institute of Pathology, Helios Klinikum, Bad Saarow, Germany; 5 Department of Urology, Cottbus, Germany

## Abstract

**Background:**

The three far-upstream element (FUSE) binding proteins (FBP1, FBP2, and FBP3) belong to an ancient family of single-stranded DNA binding proteins which are required for proper regulation of the *c-myc *proto-oncogene. Whereas it is known that *c-myc *alterations play a completely different role in various carcinomas of the urogenital tract, the relevance of FBPs is unclear.

**Methods:**

FBP1, FBP3 and *c-myc *expression was studied in 105 renal cell, 95 prostate and 112 urinary bladder carcinomas by immunohistochemistry using tissue microarrays.

**Results:**

High rates of FBP1 and FBP3 expression were observed in all cancer types. There was a concomitant up-regulation of FBP1 and FBP3 in renal cell and prostate carcinomas (p < 0.001 both). *C-myc *expression was detectable in 21% of prostate, 30% of renal and 34% of urothelial carcinomas. Interestingly, strong FBP1 and FBP3 expression was associated with *c-myc *up-regulation in clear cell renal cell carcinomas (p < 0.001 and 0.09 resp.), but not in bladder or prostate cancer.

**Conclusion:**

The correlation between FBP1/FBP3, *c-myc *and high proliferation rate in renal cell carcinoma provides strong *in viv*o support for the suggested role of FBP1 and FBP3 as activators of *c-myc*. The frequent up-regulation of FBP1 and FBP3 in urothelial and prostate carcinoma suggests that FBPs also have an important function in gene regulation of these tumors.

## Background

The three far-upstream element (FUSE) binding proteins (FBP1, FBP2, and FBP3), encoded by different genes, comprise an ancient family of single-strand DNA-binding proteins which have different functions in gene regulation. Though the FBP1, FBP2, and FBP3 genes are located on different chromosomes in mice as well as in humans, their primary sequences are highly related [1-3]. The genes encoding FBP1 and FBP3 are located on chromosomes 1p31.1 and 9q34.11, respectively. FBP1 (FBP) is designated the family progenitor. Besides regulating the transcription of the c-*myc *proto-oncogene [4-6], the FBP family has been shown to bind a variety of RNAs, therefore, FBPs are likely to be multifunctional.

The far upstream element (FUSE) of the human *c-myc *proto-oncogene stimulates expression of *c-myc *in undifferentiated cells. FBP1, FBP2, and FBP3 are single-strand DNA-binding proteins that recognize FUSE. They posses all features of conventional transcription factors. The FBPs each bind sequence-specifically to only one strand of the far upstream element (FUSE; originally identified upstream of the *c-myc *promoter), and each has potent activation domains [1]. We recently have shown that FBP is required for proper regulation of the *c-myc *proto-oncogene [6,7]. In the absence of FBP, which binds to the single-stranded FUSE, the remainder of the set fails to sustain endogenous *c-myc *expression. A dominant-negative FBP arrests cellular proliferation and extinguished native *c-myc *transcription [4]. Expression of FBP is regulated during differentiation of several tissues [8]. FBP is present in undifferentiated, but not differentiated cells of the human pro-monomyeloleucocytic cell line HL60. Expression of FBP mRNA declines upon differentiation, suggesting transcriptional regulation of FBP [9]. In addition, ubiquitination and degradation of FBP, mediated by p38, leads to down-regulation of *c-myc*, which is required for differentiation of functional alveolar type II cells [10].

The *c-myc *proto-oncogene, coding for a basic helix-loop-helix leucine zipper (bHLHZ) transcription factor, is involved in the regulation of about 10–15% of all genes – not only of class II, but also genes of class I and III, making *c-myc *a master regulator for central cellular processes such as proliferation, differentiation, apoptosis, growth and cell death. It is involved in the tumorigenesis of many human tumors [11,12] including urogenital carcinomas. Alterations of the *c-myc *genomic region are well documented for prostate cancer [13-15] as well as bladder cancer [16]. In contrast, genomic alterations of *c-myc *are mostly subordinate for cell renal carcinoma with the exception of papillary renal cancer [17-19].

Here, we have analyzed the expression of FBP1 (as the family progenitor and a moderate transcriptional activator) and FBP3 (as the strongest transcriptional activator of this family, [20]) as well as *c-myc *in renal cell carcinomas (RCC), prostate (PCA) and urothelial cancers of the urinary bladder. We found that FBP1 as well as FBP3 are more frequently expressed in prostate and bladder cancer than in renal cancer. In addition, a positive correlation between levels of FBP1, FBP3 and c-Myc was exclusively detectable in RCC.

## Methods

### Patients

#### Prostate carcinoma

Paraffin blocks from 95 prostatectomy specimens were retrieved from the archives of the Institute of Pathology, Charite Campus Mitte, Berlin, Germany. Forty-four cases were pT2, 50 cases were pT3, one case was pT4. Twenty-four cases (25%) had a Gleason score (GS) of 2–6, 39 cases (41%) had a GS of 7 and 31 (43%) cases had a GS of 8–10 (one missing due to anti-androgenic therapy). Median follow up time concerning PSA values was 43 months (range 3–180 months). Median patient age was 61 years.

#### Bladder urothelial carcinoma

We enclosed 147 patients with newly diagnosed primary non-invasive (pTa) papillary bladder cancer who underwent transurethral surgical resection (TUR) at the Carl-Thiem Hospital Cottbus, Germany, between 1997 and 2004. Grading according to WHO 1973 and WHO 2004 was done retrospectively (G.K.). Median follow up time for patients without disease progression was 53 months. Sixty-three patients (42.6%) suffered a histologically confirmed disease recurrence.

#### Renal cell carcinoma

Tumor tissue of 104 adult patients with RCC undergoing radical nephrectomy at the Department of Urology of the University Hospital Charité, Berlin, Germany between July 2003 and January 2006 was analyzed. According to the histological type, these included 83 clear cell, 16 papillary, and five chromophobe renal cell carcinomas, respectively. Tumor stage and classification were established according to the 2002 TNM System and the 2004 WHO Classification. Tumor pT-status was as follows: 53 pT1 (51%), three pT2 (3%), 45 pT3 (43%) and three pT4 (3%). According to Fuhrman, 11 tumors were G1, 73 tumors G2 and 20 tumors G3. Twenty-one tumors were pM1.

### TMAs

Tissue microarrays (TMA) with 109 renal cell carcinomas, 95 prostate and 147 urinary bladder cancers, respectively, have been constructed as described [21]. Suitable areas for tissue retrieval were marked on H&E-stained sections, punched out of the paraffin block (1.5 mm punch diameter for prostate and bladder cancer, 0.6 mm punch diameter for renal cell cancer), and inserted into a recipient block using a tissue arrayer (Beecher Instruments, Woodland, USA). The tissue array was cut without any sectioning aiding tapes, and sections (2 μm tick) were mounted on Superfrost slides (Menzel-Gläser, Braunschweig, Germany).

### Immunohistochemistry

**Figure 1:**
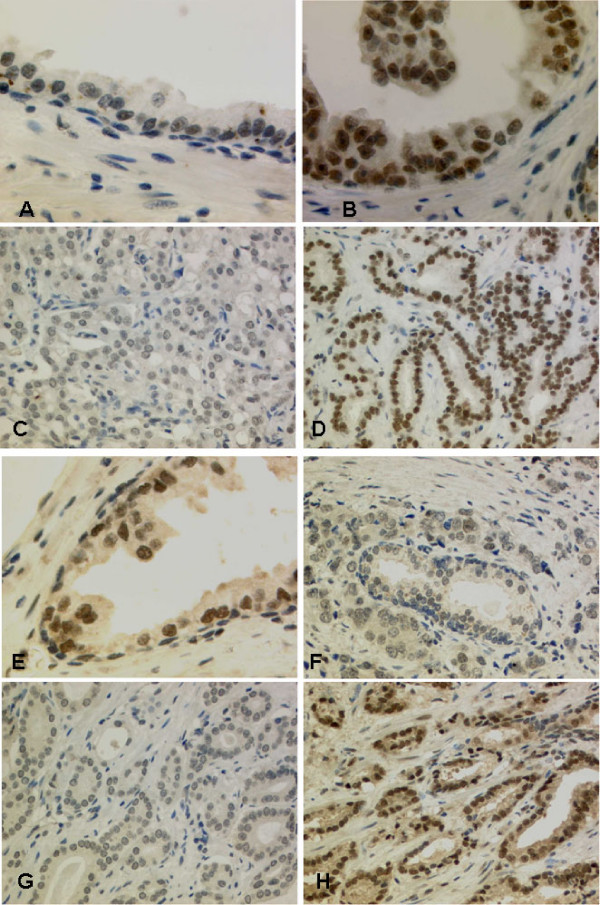
**Expression of FBP1 and FBP3 in normal prostate and prostate cancer**. A) Normal prostatic epithelium with a strong nuclear expression of FBP1, basal cells are mostly devoid of FBP1 expression (40×). B) Prostatic intraepithelial neoplasia (PIN) also shows a strong nuclear signal for FBP1 (40×). C) Prostate cancer with weak FBP1 expression (20×). D) Prostate cancer with strong FBP1 expression (20×). E) Normal prostatic epithelium also reveals a strong nuclear expression of FBP3, basal cells are again mostly negative for FBP3 (40×). F) Normal gland (central), surrounded by invasive carcinoma with equal FBP3 expression (20×). G) Prostate cancer with low FBP3 expression (20×). H) Prostate cancer with high FBP3 expression (20×).

**Figure 2:**
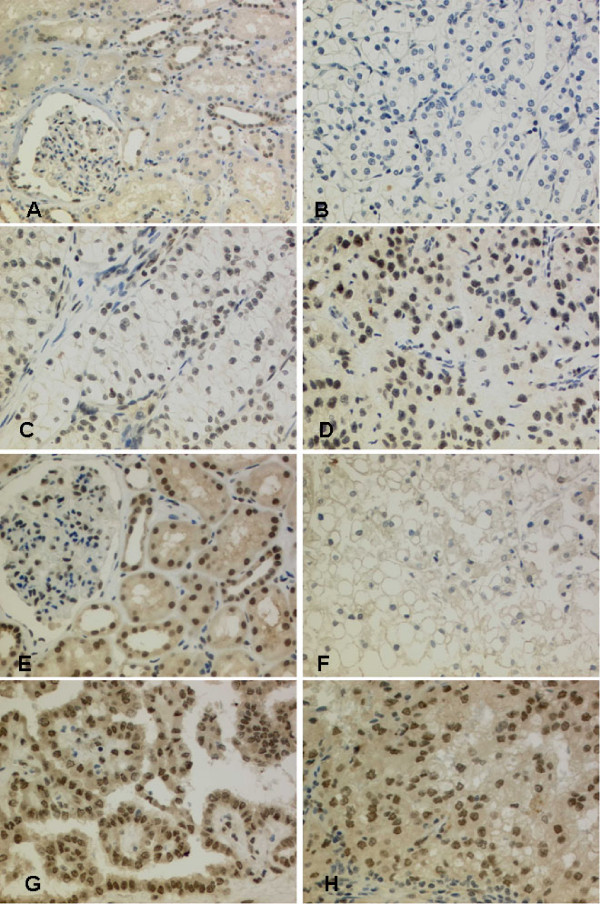
**FBP1 and FBP3 expression in normal kidney and renal cancer**. A) Normal renal tissue shows a moderate FPB1 expression in epithelia of distal tubules, whereas proximal tubules show no immunoreactivity. Podocytes and endothelia of the Bowman capsule are also FBP1 positive (20×). B) ccRCC lacking FBP1 expression (20×).C) ccRCC with moderate FBP1 immunoreactivity (20×). D) Example of a strong FBP1 expression in a ccRCC, 20×. E) Normal renal tissue with a strong FBP3 expression in epithelia of proximal and distal tubules (40×). F) FBP3-negative ccRCC (20×). G) FBP3 positive papillary RCC (20×). H) ccRCC with strong FPB3 expression (20×).

**Figure 3:**
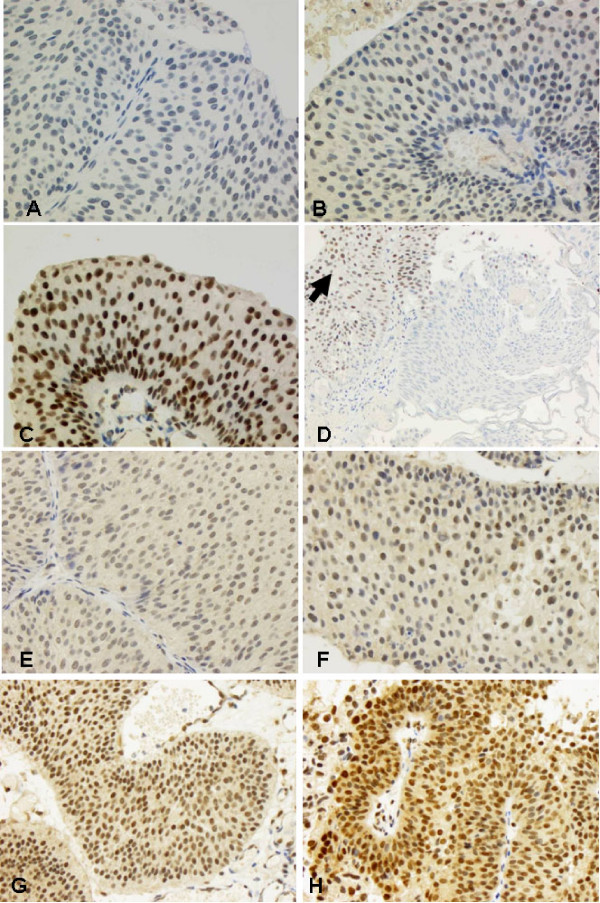
**FBP1 and FBP3 expression in normal urothelium and cancer of the urinary bladder**. Papillary bladder cancer with weak (A), with moderate (B), and with strong (C) expression of FBP1 (all 20×). D) Illustration of the heat sensitivity of the FBP1 epitope, which is not detectable in coagulated areas (centrally), whereas the signal is well retained at the periphery (arrow). Papillary bladder cancer with weak (E, 20×), moderate (F, 40×; G, 20×)), and with a strong (H, 40×) expression of FBP3, respectively.

**Figure 4:**
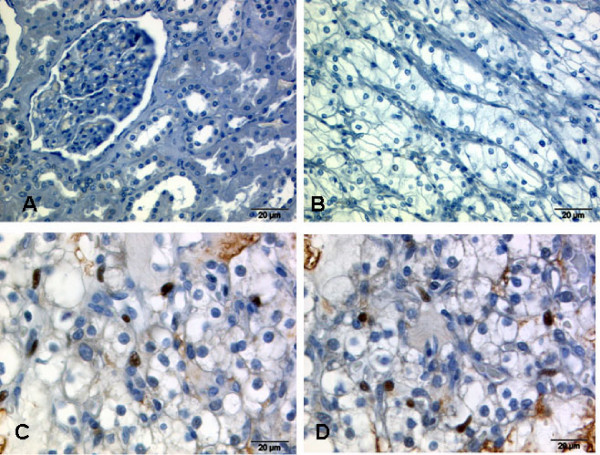
**Expression of c-Myc in normal kidney and renal cancer**. In non-tumor kidney tissue, no positive staining was found for c-Myc (A, 40×), and most renal cell carcinomas as were negative (B, 40×). Positive cases revealed staining for c-Myc only in a proportion of tumor cells (C, 40×).

TMA sections were de-paraffinized in xylene and gradually hydrated. All TMAs were analyzed with the Ventana Benchmark automated staining system (Ventana Medical Systems, Tucson, AZ) using Ventana reagents for the entire procedure. For immunostaining, commercially available antibodies against FBP1 (C-20, sc-11101, Santa Cruz Biotechnology, Heidelberg, Germany), FBP3 antibodies (E-15, sc-11104, Santa Cruz Biotechnology, Inc.), Ki-67 (clone MIB1, Dianova, Hamburg, Germany), and c-Myc (mouse monoclonal, 9E11, Novocastra Laboratories Ltd, Newcastle upon Tyne, UK) were used and adjusted to the Ventana Benchmark (Ventana Medical Systems, Tucson, AZ) system. Titrations and initial reactivity assessment were performed on TMAs containing multiple tissues of different histogenetic origin. Sections were treated with and without a heat-based steamer using various antigen different retrieval solutions (citrate buffer, EDTA buffer, cell conditioning solution). Clear nuclear staining was achieved at a dilution of 1:100 (concentration, 2 μg/mL) for FBP1 and FBP3 (Figure 1, 2, 3), 1:400 for c-Myc, and 1:1000 for Ki-67, respectively, which was used for all analysis (Figure 4). Primary antibodies were detected using the iVIEW DAB detection kit and the signal enhanced with the amplification kit. Slides were counterstained with hematoxylin, dehydrated and mounted.

With this protocol, a nearly exclusively nuclear (FBP1) or predominantly nuclear (FBP3) staining product was identified, in line with what was observed before in cell lines by transmission and confocal fluorescence microscopy [20,22]. For both, FBP1 and FBP3, only nuclear staining was assayed. Intensity of staining was scored in a semi-quantitative approach as score 0 (none to very faint), score 1 (weak), score 2 (moderate), or score 3 (strong).

For c-Myc, a stringent evaluation protocol with high dilution of antibody was used, and only a nuclear signal was interpreted as positive staining (score 0: negative, or score 1: positive). For Ki-67, percentage of positive tumor cells was recorded for correlation analysis. For statistical evaluation of Ki-67 in RCC, the Ki-67 count was dichotomized in low (< 1%) versus high rates (> 1%) of expression.

### Statistics

Statistical Product and Service Solutions (SPSS) software 15.0 for windows (SPSS Inc., 1989–1999) was used to calculate statistics. Bivariate correlations were calculated according to Spearman. In crosstabs, Fishers exact test, Chi square test and Chi square test for trends were applied. Results were considered statistically significant if *p*-values were 0.05.

## Results

### FBP1 and FBP3 expression in normal tissues

So far, immunohistochemisty for FBP1 and FBP3 in human tissues has not been reported. Therefore, we first sought to evaluate immunostaining with the antibodies used in this study on TMAs containing normal and tumor samples of different histogenetic origin. For this, a staining pattern corresponding to what has been reported before in cell lines by transmission and confocal fluorescence microscopy [20,22] was regarded as specific: a nearly exclusively nuclear staining for FBP1, and an at least predominantly nuclear staining product in the cases of FBP3. Indeed, for both transcription factors, immunohistochemistry revealed a nuclear signal in normal kidney, prostate and urothelium. For FBP3, a weak cytoplasmic reactivity was also detectable. Tubular epithelium of the nephron revealed a distinct differential staining pattern of FBP1 or FBP3: distal tubules showed a moderate FPB1 expression, whereas proximal tubules were negative for FBP1 and FBP3. Podocytes and endothelia of the Bowman capsule were FBP1 and FBP3 positive (Figure 2A). FBP3 expression was found in most epithelia of proximal and distal tubules (Figure 2E). FBP1 and FBP3 respectively were expressed in normal urothelium of the urinary bladder.

### FBP1 and FBP3 expression in prostate cancer

Expression of FBP1 and FBP3 was detectable in luminal epithelia of normal glands, prostatic intraepithelial neoplasia (PIN; Figure 1B) as well as adenocarcinomas of the prostate. FBP1 was interpretable in 91 of 95 tissue cores on the TMA. FBP3 was interpretable in all tissue cores. All tumors showed at least a weak FBP1 and FBP3 expression. Twelve tumors revealed a weak expression (10.9%) (Figure 1C), 50 tumors a moderate expression (45.5%), and 29 a strong expression of FBP1 (26.4%) (Figure 1D). For FBP3, 18 tumors revealed a weak expression (16.4%) (Figure 1G), 48 tumors a moderate expression (43.6%), and 29 a strong expression of FBP3 (26.4%)(Figure 1H). For statistical analysis, weak and moderate FBP expression was lumped (low) and opposed to strong expression (high).

A strong positive association was found between the expression intensities of FBP1 and FBP3, respectively. Expression of FBP1 or FBP3 was not associated with clinico-pathological variables (Table 1).

**Table 1:** Associations between FBP1 and -3 and clinical parameters in prostate cancer (low, high expression: for definition see text).

	**FPB1**				**FBP3**			
	
	n	low n (%)	high n(%)	p-value	n	low n (%)	high n(%)	p-value
								
**pT2**	42	29 (69.0)	13 (31.0)	n.s.	44	31 (70.5)	13 (29.5)	n.s.
**pT3/4**	49	33 (67.3)	16 (32.7)		51	35 (68.6)	16 (31.4)	
**Gleason score 2–6**	23	16 (69.6)	7 (30.4)	n.s.	24	14 (58.3)	10 (41.7)	n.s.
**Gleason score 7–10**	67	46 (68.7)	21 (31.3)		70	51 (72.9)	19 (27.1)	
**PSA relapse**	48	34 (70.8)	14 (29.2)	n.s.	49	32 (65.3)	17 (34.7)	n.s.
**No PSA relapse**	43	28 (65.1)	15 (34.9)		46	34 (73.9)	12 (26.1)	
**FBP1 low**	62				62	48 (77.4)	14 (22.6)	0.017
**FBP1 high**	29				29	15 (51.7)	14 (48.3)	
**FBP3 low**	63	48 (76.2)	15 (23.8)	0.017	66			
**FBP3 high**	28	14 (50.0)	14 (50.0)		29			

n.s.: not significant

### FBP1 and FBP3 expression in renal cancer

Staining of FBP1 was evaluable in 104 renal cell carcinomas. Of these, 19 tumors were FBP1 negative (17.4%) (Figure 2B). 49 tumors revealed a weak expression (45.0%), 31 tumors a moderate expression (28.4%) (Figure 2C), and 5 a strong expression of FBP1 (4.6%) (Figure 2D). Staining of FBP3 was analyzable in a total of 105 RCCs, with 12 tumors being negative for FBP3 (11.0%) (Figure 2F). 47 tumors revealed a weak expression (43.1%), 37 tumors a moderate expression (33.9%), and nine a strong expression of FBP3 (8.3%) (Figure 2G&2H). Negative and weak versus moderate and strong FBP immunoreactivity was lumped (low *vs. *high) for statistical analysis. A strong positive association was found between the expression intensities of FBP1 and FBP3, respectively (Table 2). Importantly, there was an association between high FBP expression levels and the renal tumor subtype. High FBP1 expression was more frequent in papillary RCC (68.8%) than in clear cell RCC (30.1%; p < 0.003). A similar trend was observed for FBP3 (p = 0.052). In clear cell RCC, FBP3 expression was associated with tumor stage: high FBP3 expression was less frequent in pT3/4 stage (25.6%%) than in pT1/2 stage (52.5%; p = 0.014). There were no other correlations between the expression of FBP1 or FBP3 and clinicopathological parameters (Table 2).

**Table 2:** Associations between FBP1 and -3 and clinical parameters in RCC (low, high expression: for definition see text).

		**FPB1**				**FBP3**			
		
		n	low n (%)	high n (%)	p-value	n	low n (%)	high n (%)	p-value
									
**Histology**	**clear cell**	83	58 (69.9)	25 (30.1)	0.003^#^	83	51 (61.4)	32 (38.6)	0.052^#^
	**papillary**	16	5 (31.3)	11 (68.8)		17	5 (29.4)	12 (70.6)	
	**chromo-phobe**	5	5 (100.0)	0 (0)		5	3 (60.0)	2 (40.0)	
**pT** Stage*	**pT1/2**	40	30 (75.0)	10 (25.0)	n.s.	40	19 (47.5)	21 (52.5)	0.014
	**pT3/4**	43	28 (65.1)	15 (34.9)		43	32 (74.4)	11 (25.6)	
Grade*	**1/2**	66	48 (72.7)	18 (27.3)	n.s.	66	43 (65.2)	23 (34.8)	n.s.
	**3/4**	17	10 (58.8)	7 (41.2)		17	8 (47.1)	9 (52.9)	
**FBP1**	**low**	58				58	41 (70.7)	17 (29.3)	0.013
	**high**	25				25	10 (40.0)	15 (60.0)	
**FBP3**	**low**	51	41 (80.4)	10 (19.6)	0.013	51			
	**high**	32	17 (53.1)	15 (46.9)		32			

n.s.: not significant

* only clear cell RCC

^# ^Chi square test

### FBP1 and FBP3 expression in urothelial cancer of the urinary bladder

FBP1 immunohistochemistry was evaluable in 112 urothelial carcinomas. Thirty-seven revealed a weak expression (33%) (Figure 1A), 56 tumors a moderate expression (50%) (Figure 1B), and 19 a strong expression of FBP1 (17%) (Figure 1C). FBP3 was analyzable in 102 bladder cancer cases: 25 tumors revealed a low expression (25%) (Figure 3E), 57 tumors showed a moderate expression (57%) (Figure 3F&3G), and 20 a strong expression of FBP3 (20%)(Figure 3H). In contrast, to PCAs and RCCs, the co-expression of FBP1 and FBP3 failed statistical significance but showed a clear trend with 53 tumors that had high levels of FBP1 and FBP3 (p = 0.067). Additionally, high FBP1 expression was significantly more frequent in low grade (71.9%) than in high grade (47.8%) tumors (p = 0.029)(Table 3).

**Table 3:** Associations between FBP1 and -3 and tumor grade in non-invasive bladder cancer RCC (low, high expression: for definition see text).

		**FPB1**				**FBP3**			
		
		n	low n (%)	high n (%)	p-value	n	low n (%)	high n (%)	p-value
									
**Grade** **(WHO 1973)**	**G1**	27	7 (25.9)	20 (74.1)	n.s.*	24	4 (16.7)	20 (83.3)	n.s.*
	**G2**	80	27 (33.8)	53 (66.3)		74	19 (25.7)	55 (74.3)	
	**G3**	5	3 (60.0)	2 (40.0)		4	2 (50.0)	2 (50.0)	
**Grade (WHO 2004)**	**Low grade**	89	25 (28.1)	64 (71.9)	0.029*	81	20 (24.7)	61 (75.3)	n.s.*
	**High grade**	23	12 (52.2)	11 (47.8)		21	5 (23.8)	16 (76.2)	
**FBP1**	**low**	37				36	12 (33.3)	24 (66.7)	0.067
	**high**	75				64	11 (17.2)	53 (82.8)	
**FBP3**	**low**	23	12 (52.2)	11 (47.8)	0.067*	25			
	
	**high**	77	24 (31.2)	53 (68.8)		77			

n.s.: not significant

* Chi square test for trends

### c-Myc and FBP expression

To determine c-Myc protein expression in relation to FBP expression levels, we developed a stringent protocol for c-Myc immunohistochemistry. Using this protocol, there was no c-Myc staining in non-tumoral tissues (Figure 4A). In contrast, c-Myc was detectable in about one third of all tumors: 21.3% of prostate carcinomas, 29.9% of renal cell carcinomas, and 33.9% of urothelial carcinomas were positive.

In prostate cancer, c-Myc did neither correlate to pT stage, Gleason score and preoperative PSA levels nor to FBP1/FBP3 expression or Ki-67 fraction.

In bladder cancer, c-Myc correlated to tumor grading (WHO 2004: correlation coefficient 0.267, p = 0.003), but did not correlate to FBP1 or FBP3 expression or Ki-67 fraction.

In renal cell cancer, no correlations of c-Myc with pT stage, M stage, grading or histology were found. On stratified analysis, a significant association of c-Myc and FBP1 (p = 0.001) expression (Table 4. Figure 4B–D) was apparent in clear cell RCC. 62.5% of clear cell RCC with high FBP1 were c-Myc positive. In contrast, c-Myc expression was only seen in 20.7% of clear cell RCC with low FBP1 expression. Such a trend was also observed for FBP3 (p = 0.09). Additionally, a highly significant association between high levels of FBP1 and high tumor cell proliferation rate (Ki-67) (p = 0.001) was noted. This is in sharp contrast to papillary renal cell carcinomas. In this tumor subtype, no association between FBP1/FBP3 expression and c-Myc or Ki-67 fraction was seen (data not shown).

**Table 4:** Associations between FBP1, FBP-3, c-Myc, and proliferation rate (Ki-67) in clear cell RCC

		**c-myc**				**Ki-67**			
		n	neg. n (%)	pos. n (%)	p-value	n	low n (%)	high n (%)	p-value
**FBP1**	**low**	58	46 (79.3)	12 (20.7)	0.001	58	49 (84.5)	9 (15.5)	0.001
	**high**	24	9 (37.5)	15 (62.5)		25	5 (20.0)	20 (80.0)	
**FBP3**	**low**	51	38 (74.5)	13 (25.5)	0.09	51	36 (70.6)	15 (29.4)	n.s.
			
	**high**	31	17 (54.8)	14 (45.2)		32	18 (56.3)	14 (43.8)	

* Chi square test for trends

## Discussion

The single-stranded binding proteins FBP1 and FBP3 are involved in the regulation of many cellular processes, such as gene expression and differentiation of several tissues [4,7,8,10]. With this, FBPs obviously are potential targets of a malignant cell transformation. The major aim of this study was to evaluate FBP1 and FBP3 expression in urogenital tumors and to elucidate the relationship to c-Myc expression. Our study showed different expression levels of the single-stranded proteins in renal cancer subtypes, as well as in bladder and prostate cancer. Further, we demonstrate a significant correlation between FBP1 and c-myc expression in clear cell renal cancer, whereas there was no such correlation in renal papillary, prostate and bladder cancer.

In previous studies, we and others have investigated FBP1 and FBP3 expression in human cell lines by either immunofluorescence studies or fusion protein experiments in cell culture systems [6,20,22,23]. Here, we have extended FBP expression analysis to primary non-neoplastic and neoplastic human tissues using immunohistochemistry. Remarkably, the subcellular localization patterns of FBP1 and FBP3 with a nearly exclusive (in case of FBP1) or at least predominant localization (in case of FBP3) in the nucleus we observed by means of immunohistochemistry were similar to what has been described before. The overlapping data on subcellular localization patterns from the previously reported cell culture studies with our data indicated that the immunostaining for FBP1 and FBP3 were report here is a reliable means of studying these proteins. Furthermore, our observations in primary human tissues confirm previous data ascribing FBPs mainly a function as transcription factors.

Since previous studies on FBPs revealed an important role of these proteins in the regulation of the *c-myc *oncogene, we studied c-Myc protein levels. So far, very different immunohistochemical staining patterns and intensities are reported for c-Myc. Therefore, we deemed using a stringent protocol for c-Myc immunohistochemistry indispensible for reliable c-Myc staining. With this, we have observed no staining in non-tumoral tissues, and nuclear staining in roughly one third of all tumors. The results obtained with our immunohistochemical staining protocol are highly congruent with these reported findings. Therefore, we regard our c-Myc staining as specific.

With this immunohistochemical procedure, we found different patterns between FBP1, FBP3 and *c-myc *expression in the different tumor types. Clear cell RCC was the only tumor type with a positive correlation of FBP1 and FBP3 expression levels with c-Myc expression. This may recapitulate the view that FBPs are activators of *c-myc in renal cancer*. While *c-myc *alterations on the genomic level have been reported in prostate [13-15] and bladder cancer [16], DNA copy number changes of *c-myc *have been identified only in papillary, but not in clear cell RCC [17-19]. It is tempting to speculate that c-myc expression in bladder, prostate and papillary renal cancer is rather due to *c-myc *copy number alterations, than a consequence of FBP overexpression, whereas clear cell RCC without *c-myc *DNA copy number alterations require FBPs for c-Myc regulation. We have recently shown that an intact chromosomal architecture of the *c-myc *regulator region is required for proper regulation of the *c-myc *gene by the FBPs [6,7,23]. The strong positive correlation between FBP1 expression and c-Myc in clear cell renal cell carcinomas is perfectly in line with the well established function of FBP1 as a transcriptional activator of *c-myc*. In papillary renal cell carcinomas with *c-myc *gene amplifications [17] one may expect a disruption of the FUSE/FBP-system. As a consequence, FBP1 expression may trigger increased proliferation in clear cell, but not in papillary RCC. This would explain our finding that the Ki-67 labeling index is related to FBP1 expression only in clear cell RCC, but not in other tumor subtypes.

We found a high degree of co-expression of FBP1 and FBP3. Although the FBP1 and FBP3 genes are located on different chromosomes in humans (1p31.1 and 9q34.11, respectively) they show a strikingly parallel expression pattern through different tumor entities and tumor samples within an entity. In a recent study on the three FBPs, comparing their intrinsic activation and repression, subcellular localization, and in vivo targets, it has been shown that they usually cooperate to regulate the expression profiles among a set of common targets [20]. Our data are consistent with such a high degree of FBP1 and FBP3 co-regulation and support the notion that even in neoplastic tissues, where regulatory nexus are expected to be disturbed, FBPs act synergistically. Urothelial tumors provide an exception to the tendency of this co-regulation of the different FBPs. The most frequent genetic alteration in transitional cell carcinoma of the urinary bladder is particular loss of chromosome 9 [24]. The partial 9q losses also involved 9q33–9q34 [25]. Therefore, FBP3 on 9q34.11 may also be a frequent target of 9q losses. This would explain the disrupted co-expression of FBP1 and FBP3 in urothelial tumors.

## Conclusion

This is the first comprehensive study to investigate expression patterns of the *c-myc *regulators FBP in carcinomas of the urinogenital tract. FBP1 and FBP3 were shown to be co-expressed in most tumor entities. Our data show co-expression of FBPs and *c-myc *in vivo, which suggests a potential role of FBP1 and FBP3 as an activator of *c-myc *in clear cell RCC.

## Abbreviations

FBP: FUSE binding protein; FUSE: far-upstream element; RCC: renal cell carcinoma; TMA: tissue microarrays (TMA).

## Competing interests

The authors declare that they have no competing interests.

## Authors' contributions

Design of the study: AW, HM, GK. Colleting of patients data: MJ, BO, KS, SG, MM, HAM. Implementation of immunostaining: AW, SB. Evaluation of immunostaining and data analysis: AW, IK, GK. Writing of the manuscript: AW, HM, GK.

## Pre-publication history

The pre-publication history for this paper can be accessed here:


